# Low Serum High Density Lipoprotein Cholesterol Concentration is an Independent Predictor for Enhanced Inflammation and Endothelial Activation

**DOI:** 10.1371/journal.pone.0116867

**Published:** 2015-01-23

**Authors:** Wan Nor Hanis Wan Ahmad, Farah Sakri, Atiqah Mokhsin, Thuhairah Rahman, Nadzimah Mohd Nasir, Suraya Abdul-Razak, Mazapuspavina Md Yasin, Aletza Mohd Ismail, Zaliha Ismail, Hapizah Nawawi

**Affiliations:** 1 Centre for Pathology Diagnostic and Research Laboratories (CPDRL), UniversitiTeknologi MARA (UiTM), Sungai Buloh Campus, Sungai Buloh, Selangor, Malaysia; 2 Cluster for Pathology and Laboratory Medicine, UniversitiTeknologi MARA (UiTM), Sungai Buloh Campus, Sungai Buloh, Selangor, Malaysia; 3 Primary Care Medicine Discipline, UniversitiTeknologi MARA (UiTM), Sungai Buloh Campus, Sungai Buloh, Selangor, Malaysia; 4 Discipline of Population Health and Preventive Medicine, Faculty of Medicine, UniversitiTeknologi MARA (UiTM), Sungai Buloh Campus, Sungai Buloh, Selangor, Malaysia; Heart Research Institute, AUSTRALIA

## Abstract

**Background:**

Inflammation, endothelial activation and oxidative stress have been established as key events in the initiation and progression of atherosclerosis. High-density lipoprotein cholesterol (HDL-c) is protective against atherosclerosis and coronary heart disease, but its association with inflammation, endothelial activation and oxidative stress is not well established.

**Objectives:**

(1) To compare the concentrations of biomarkers of inflammation, endothelial activation and oxidative stress in subjects with low HDL-c compared to normal HDL-c; (2) To examine the association and correlation between HDL-c and these biomarkers and (3) To determine whether HDL-c is an independent predictor of these biomarkers.

**Methods:**

422 subjects (mean age±SD = 43.2±11.9years) of whom 207 had low HDL-c concentrations (HDL-c <1.0mmol/L and <1.3mmol/L for males and females respectively) and 215 normal controls (HDL-c ≥1.0 and ≥1.3mmol/L for males and females respectively) were recruited in this study. The groups were matched for age, gender, ethnicity, smoking status, diabetes mellitus and hypertension. Fasting blood samples were collected for analysis of biomarkers of inflammation [high-sensitivity C-reactive protein (hsCRP) and Interleukin-6 (IL-6)], endothelial activation [soluble Vascular Cell Adhesion Molecule-1 (sVCAM-1), soluble Intercellular Adhesion Molecule-1 (sICAM-1) and E-selectin)] and oxidative stress [F2-Isoprostanes, oxidized Low Density Lipoprotein (ox-LDL) and Malondialdehyde (MDA)].

**Results:**

Subjects with low HDL-c had greater concentrations of inflammation, endothelial activation and oxidative stress biomarkers compared to controls. There were negative correlations between HDL-c concentration and biomarkers of inflammation (IL-6, *p* = 0.02), endothelial activation (sVCAM-1 and E-selectin, *p* = 0.029 and 0.002, respectively), and oxidative stress (MDA and F_2_-isoprostane, *p* = 0.036 and <0.0001, respectively). Multiple linear regression analysis showed HDL-c as an independent predictor of IL-6 (*p* = 0.02) and sVCAM-1 (*p*<0.03) after correcting for various confounding factors.

**Conclusion:**

Low serum HDL-c concentration is strongly correlated with enhanced status of inflammation, endothelial activation and oxidative stress. It is also an independent predictor for enhanced inflammation and endothelial activation, which are pivotal in the pathogenesis of atherosclerosis and atherosclerosis-related complications.

## Background

High density lipoprotein cholesterol (HDL-c) is the smallest and densest of the five major lipoprotein particles that function to collect, esterify and transport cholesterol from the peripheral tissues to the liver. HDL-c is considered a good cholesterol due to its crucial function in reverse cholesterol transport (RCT), the mechanism by which cholesterol is taken out from cells and transported to the liver for remodeling and disposal [1]. It was established that low HDL-c concentration can be caused by several factors such as smoking, physical inactivity and dietary intake. Low HDL-c concentration is an independent risk factor for coronary heart disease (CHD) and used as a criteria in the Framingham Risk Scoring (FRS) calculation to determine CHD risk of individual patients [2]. Epidemiology studies affirmed that HDL-c concentration is inversely proportional to incidence of CHD and peripheral vascular diseases (PVD) [3–5]. CHD and other cardiovascular diseases mainly result from underlying atherosclerosis; which is a complex disease process involving indolent inflammation, endothelial activation and oxidative stress [6].

Inflammation, endothelial activation and oxidative stress contribute to the development and progression of atherosclerosis. Several biomarkers have been established to reflect the status of these processes. High sensitivity C-reactive protein (hsCRP) and Interleukin-6 (IL-6) represent biomarkers of inflammation [7], while endothelial activation is reflected by the various adhesion molecules such as soluble vascular cell adhesion molecule-I (sVCAM-1), soluble intracellular cell adhesion molecule-I (sICAM-1) and E-selectin [8]. These soluble adhesion molecules serve as surrogate markers for increased vascular endothelial cells expression of adhesion molecules (CAMs) which in turn reflect the activation of these cells [9]. HDL-c concentration has been shown to down-regulate the expression of endothelial cell adhesion molecules [10, 11]. Oxidized low density lipoprotein (ox-LDL), Malondialdehyde (MDA) [12], and F_2_-isoprostane have been used as biomarkers for oxidative stress [13].

HDL-c particles demonstrate multiple anti-atherogenic activities, mainly in the reverse cholesterol transport (RCT) which is the process of removing excessive free cholesterol from the arterial wall to the liver for excretion. They also possess anti-oxidant, anti-inflammatory, anti-apoptotic, anti-thrombotic, anti-infective and vasodilatory effects. The initial step of RCT involves efflux of free cholesterol from peripheral blood cells, particularly macrophages via ATP-binding Cassette Transporter A1 (ABCA1). ApoA1 which lies on the surface of HDL particles activates the enzyme lecithin-cholesterol acyltransferase (LCAT), which then allows the latter to esterify the accepted free cholesterol to cholesterol ester, transforming HDL_3_ particles into HDL_2_ particles,the mature form of HDL-c [14]. Then, Scavenger Receptor B1 (SRB1) acts as a mediator and selectively takes up the accumulated cholesteryl ester from matured HDL-c into the liver for excretion [15].

High concentration of HDL-c was shown to protect against the development of atherosclerosis, where 1 mg/dL increment of HDL-c concentration was associated with 2% and 3% CHD risk reduction in men and women, respectively [16]. Various studies have reported that HDL-c exhibits anti-inflammatory and anti-oxidant properties. Ansell et al showed the anti-inflammatory properties of HDL-c, where higher concentration of HDL-c is associated with lower hsCRP concentration [17]. Another study reported that HDL-c particles exhibit normalized anti-atherogenic properties mainly in reverse cholesterol transport and claimed that HDL-c is a promising therapeutic strategy for treatment of dyslipidaemia, inflammation as well as premature atherosclerosis [18]. Low HDL-c has been reported among diabetes mellitus (DM) patients and it was observed that the anti-atherogenic properties of HDL-c appear to improve diabetic control [19].

Recently, considerable controversies were raised concerning whetheror not HDL-c concentration reflects HDL-c functions, and whether it is even protective, as has been reported in several previous studies [20–22]. Futhermore, although significant animal studies and clinical trials supported the atheroprotective role of HDL-c, most of these were in context of marked changes in other plasma lipids [23]. Despite numerous reports on the association between HDL-c concentration and biomarkers of inflammation, endothelial activation and oxidative stress, there is scarcity of such data in Asian countries, particularly among the Malaysian population. In addition, majority of studies investigating the associations between HDL-c concentration and status of inflammation, endothelial activation and oxidative stress biomarkers, did not did not exclude subjects on medications such as lipid-lowering, anti-hypertensive and/or anti-diabetic drugs, which are potential confounding factors to inflammation, endothelial activation and oxidative stress [24, 25]. In addition, it is unclear whether HDL-c concentration is an independent predictor of these biomarkers after correcting for various confounding factors such as age, gender, ethnicity, smoking status, hypertension, diabetes, obesity [body mass index (BMI) and waist circumference (WC)], triglycerides (TG), low density lipoprotein cholesterol (LDL-c) as well as total cholesterol (TC).

Therefore, this study was aimed to (1) compare the concentrations of inflammation, endothelial activation and oxidative stress biomarkers in Malaysian subjects with low HDL-c and normal controls, (2) examine the association and correlation between HDL-c and these coronary risk biomarkers and (3) determine whether HDL-c is an independent predictor of the various biomarkers after correcting for the confounding factors.

## Methods

### Study Participants

A total of 422 subjects were recruited for this cross sectional study. The subjects consisted of 207 subjects with low HDL-c concentration (<1.0 mmol/L and <1.3 mmol/L in males and females respectively; 78 males and 129 females; age range: 18 to 65 years; mean age ± SD = 43.2 ± 11.9 years) and 215 normal controls (NC) with HDL-c concentration ≥1.0 mmol/L and ≥1.3 mmol/L in males and females respectively; 79 males and 136 females; age range: 18 to 65 years; mean age ± SD = 43.5 ± 12.1 years). Both groups were matched for age, gender and ethnicity, DM, hypertension and smoking status. The exclusion criteria for both groups were LDL-c concentration of more than 4.2 mmol/L,those on any anti-hypertensive, anti-diabetic and/or lipid lowering medications, in acute or chronic inflammatory state [e.g.: acute appendicitis, acute dermatitis, acute tonsillitis, upper respiratory tract infection (URTI)], chronic obligate autoimmune inflammatory diseases [e.g.: rheumatoid arthritis (RA) andsystemic lupus erythematosus (SLE)], have previous/current history of renal, liver or endocrine disorder and malignancy or diseases that shorten life span. The recruited subjects with hypertension and/or diabetes were either newly diagnosed and/or drug naïve with regards to anti-hypertensive and anti-diabetic medications.

### Ethics statement

All subjects provided written, informed consent and approval of the Institutional Board of Ethics Committee at Universiti Teknologi MARA (UiTM), Malaysia was obtained prior to commencement of the study.

### Demographic characteristics

A standard questionnaire was used to obtain demographic data including lifestyle risk factors (ie. smoking status), hypertension, DM, personal and family history of CHD. Anthropometric data were also collected, including height and body weight to determine BMI, WC, waist-to-hip ratio (WHR) and blood pressure (BP). Height and weight were measured by trained staff using a balance beam scale with subjects’ shoes removed. BP was measured by an automated BP reader (cuff size 12 x 33 cm, Colin press-mate, Japan), with subjects in a sitting position for at least 5 to 10 minutes of rest prior to examination. BMI was calculated as weight in kilograms divided by the square of the height in meters (kg/m^2^). WC was measured to the nearest 0.5cm using a measuring tape midway between the inferior margin of the last rib and the iliac crest in a horizontal plane. Hip circumference measurement was taken around the pelvis at the point of maximal protrusion of the buttocks and WHR was determined by dividing the subject’s waist over hip circumference.

### Sample collection and analysis for biomarkers of inflammation, endothelial activation and oxidative stress

Blood samples were collected in the morning following 10 to 12 hours of fasting. All blood samples were centrifuged at 3500 rpm for 7 minutes to extract serum and plasma samples which were kept frozen at −20°C until laboratory testing. Fasting plasma glucose was analyzed using the hexokinase enzymatic reference method on an automated analyzer. TC, TG, and HDL-c were measured by enzymatic reference methods, while hsCRP concentration was measured by turbidimetric assay on an automated analyzer (Cobas Integra 400 by Roche, Germany). LDL-c concentration was calculated using the Friedewald equation. Serum IL-6, sVCAM-1, sICAM-1 and E-selectin concentrations were measured by enzyme linked immunosorbent assay (ELISA) (eBioscience Bender MedSystems, Vienna Austria). Serum ox-LDL concentration was measured using an ELISA kit (Mercodia, Sweden). MDA concentration was measured by a method adapted from Ledwozyw et al, (1986). All absorbances were read on a microplate reader (Tecan Sapphire II, Austria). F_2_-isoprostanes concentration was analyzed by liquid chromatography-tandem mass spectrometry method on the 4000 QTRAP (Applied Biosystem, Canada) following pretreatment of the samples using diethyl ether.

### Statistical analysis

All statistical analysis was performed using SPSS software version 16.0 for Windows. Continuous data were presented as mean ± standard error of mean (SEM) while categorical data was expressed as percentage. Analysis of normality was performed using Kolmogorov Smirnov test. Differences between the two groups were tested using *t*-test for normally distributed continuous variables. Pearson’s correlation coefficient was used to analyze correlation and multiple linear regression analyses were used to determine independent predictor of the biomarkers of inflammation, endothelial activation and oxidative stress of the whole study population. All *p*-value was two-tailed and *p* <0.05 was considered as statistically significant.

## Results

### Demographic and clinical characteristics of study population

The demographic and clinical characteristics of the studied subjects are shown in Table 1. Compared to NC, subjects with low HDL-c had significantly higher BMI, WC, and TG but lower TC and LDL-c concentrations. There was no significant difference in BP and plasma glucose concentration observed between the two groups. Both groups were matched for age, gender, ethnicity, smoking status, BP, glucose concentration and proportions of hypertensive and diabetic subjects.

**Table 1:** Demographic and clinical characteristics of low HDL-c subjects and controls.

Parameters	Low HDL (n = 207)	Controls (n = 215)	*p*-value
^b^Age (years)	43.2 ± 11.9	43.5 ± 12.1	NS
ªGender (males/ females)	37.7/62.3	36.7/63.3	NS
ªEthnic (Malay/ Chinese/ Indian/ Others)	78.3/3.7/0.5/17.5	81.9/1.9/1.4/14.8	NS
ªHypertension	29.8	31.3	NS
^b^SBP (mmHg)	127.4 ± 19.3	125.4 ± 19.7	NS
^b^DBP (mmHg)	79.0 ± 11.7	78.0 ± 13.3	NS
ªDiabetes	8.0	4.8	NS
^b^Plasmaglucose (mmol/L)	6.1 ± 2.9	5.9 ± 3.0	NS
^b^BMI (kg/m^2^)	27.4 ± 5.4	24.8 ± 4.8	<0.001
ªCentral Obesity	67.2	41.0	<0.001
^b^Waist circumference (cm)	88.2 ± 10.7	81.1 ± 11.1	<0.001
ªCurrent smoker	18.9	18.8	NS
^b^Total cholesterol (mmol/L)	4.8 ± 1.4	5.5 ± 0.9	<0.001
^b^Triglycerides	2.5 ± 1.8	1.4 ± 0.8	<0.001
^b^LDL-c (mmol/L)	2.8 ± 1.1	3.4 ± 0.8	<0.001
^b^HDL-c (mmol/L)	0.8 ± 0.2	1.5 ± 0.3	<0.001

SBP, systolic blood pressure; DBP, diastolic blood pressure; BMI, Body mass index

^a^Data expressed as proportion (percentage)

^b^Data expressed as Mean±SD

NS—not significant

### Comparison of concentrations of biomarkers between subjects with low HDL-c versus controls

Individuals with low HDL-c had higher concentrations of inflammatory biomarkers compared to NC; mean ± SEM [hsCRP (2.50 ± 0.17 vs. 1.86 ± 0.16mg/L, *p* = 0.006) and IL-6 (5.68 ± 0.16 vs. 4.66± 0.12 pg/ml, *p*<0.0001)]. The low HDL-c group also exhibited a greater state of endothelial activation, as indicated by higher concentrations of sVCAM-1 [mean ± SEM (735.1 ± 40.4 vs. 577.2 ± 49.0 ng/ml, *p* = 0.002)], sICAM-1 [mean ± SEM (838.5 ± 34.2 vs. 730.6 ± 28.8 ng/ml, *p* = 0.016)] and E-selectin [mean ± SEM (41.8± 2.8vs. 28.3 ± 2.3 ng/ml, *p*<0.001)] compared to NC. As for oxidative stress biomarkers, ox-LDL and F_2_-Isoprostanes concentrations were higher in low HDL-c group; mean ± SEM [(33.7 ± 1.1 vs 29.7 ± 1.2mU/L, *p* = 0.012) and (3.40 ± 0.18 vs 2.47 ± 0.16ng/ml, *p*<0.0001)], respectively compared to NC group. There was no significant difference in MDA concentration between the two groups (See summary in Table 2).

**Table 2:** Concentrations of inflammatory, endothelial activation and oxidative stress biomarkers in subjects with low HDL-c versus controls.

Biomarkers	Low HDL-c	Normal HDL-c	*p*-value
Inflammation			
hsCRP (mg/L)	2.50 ± 0.17	1.86 ± 0.16	0.006
IL-6 (pg/ml)	5.68 ± 0.16	4.66 ± 0.12	<0.001
Endothelial Activation			
sVCAM-1 (ng/ml)	735.1 ± 40.4	577.2 ± 49.0	0.002
sICAM-1 (ng/ml)	838.5 ± 34.2	730.6 ± 28.8	0.016
E-selectin (ng/ml)	41.8 ± 2.8	28.3 ± 2.3	<0.001
Oxidative stress			
ox-LDL (mU/L)	33.7 ± 1.1	29.7 ± 1.2	0.012
MDA (nmol/ml)	1.52 ± 0.04	1.48 ± 0.09	NS
F2- Isoprostanes (ng/ml)	3.40 ± 0.18	2.47 ± 0.16	<0.001

NS—not significant

Data expressed as Mean±SEM

### Correlation between HDL-c and biomarkers of inflammation, endothelial activation and oxidative stress

Pearson's correlation analysis showed negative correlations between HDL-c concentration and biomarkers of inflammation (IL-6; *p* = 0.02, r = −0.119), endothelial activation (sVCAM-1, *p* = 0.029, r = −1.109; E-selectin, *p* = 0.002, r = −0.154) and oxidative stress (F2-isoprostane, *p* < 0.0001, r = −0.198; MDA, *p* = 0.036, r = −0.107). F_2_-Isoprostanes was strongly correlated with HDL-c concentration but not with the other biomarkers (See summary in Table 3).

**Table 3:** Correlation between concentrations of HDL-c and inflammatory, endothelial activation and oxidative stress biomarkers.

	HDL-c (mmol/L)	hsCRP (mg/L)	IL-6 (pg/ml)	sICAM-1 (ng/ml)	sVCAM-1 (ng/ml)	E-selectin (ng/ml)	ox-LDL (mU/L)	F2-isoprostane (ng/ml)	MDA (nmol/ml)
HDL-c (mmol/L)	–	−0.09	−0.119*	0.042	−0.109*	−0.154**	−0.078	−0.198***	−0.107*
hsCRP (mg/L)	−0.09	–	0.211***	0.111	0.005	0.09	0.053	0.104	−0.024
IL-6 (pg/ml)	−0.119*	0.211***	–	0.024	0.058	0.181***	0.062	0.013	−0.023
sICAM-1 (ng/ml)	0.042	0.111	0.024	–	0.162**	0.111*	0.142**	−0.029	−0.139**
sVCAM-1 (ng/ml)	−0.109*	0.005	0.058	0.162**	–	0.231***	−0.047	−0.068	0.039
E-selectin (ng/ml)	−0.154**	0.09	0.181***	0.111*	0.231***	–	−0.016	0.023	0.02
ox-LDL (mU/L)	−0.078	0.053	0.062	0.142**	−0.047	−0.016	–	−0.078	−0.157**
F2-isoprostane (ng/ml)	−0.198***	0.104	0.013	−0.029	−0.068	0.023	−0.078	–	0.03
MDA (nmol/ml)	−0.107*	−0.024	−0.023	−0.139**	0.039	0.02	−0.157**	0.03	–

Significant at,*p<0.05, **p<0.01, ***p < 0.001

### Association between low or normal HDL-c subject groups and concentration quartiles of the biomarkers

Chi square analysis showed low HDL-c and NC groups were inversely associated with the quartiles of all biomarkers except for ox-LDL. A majority of low HDL-c subjects were found in the highest quartiles of each biomarkers except for ox-LDL (See summary in Table 4).

**Table 4:** Association of HDL-c categories and quartiles of biomarkers.

Biomarkers	Low HDL-c *n*(%)	Normal HDL-c *n*(%)	*p*-value
hsCRP (mg/L)			0.001
<0.5	30 (9)	59 (17)	
0.51–1.30	35 (10)	51 (15)	
1.31–3.25	51 (15)	34 (10)	
≥3.26	48 (14)	37 (11)	
IL-6 (pg/ml)			0.002
≤4.6	77 (20)	110 (29)	
4.7–6.2	45 (12)	49 (13)	
6.3–9.0	47 (9)	32 (8)	
≥9.1	15 (4)	4 (1)	
sVCAM-1 ng/ml)			0.03
≤556	80 (20)	117 (29)	
557–796	60 (15)	40 (10)	
797–1795	41 (10)	40 (10)	
≥1476	15 (4)	7 (2)	
sICAM-1 (ng/ml)			0.016
≤650	82 (22)	108 (28)	
651–931	46 (12)	50 (13)	
932–1706	48 (13)	29 (8)	
≥1707	12 (3)	6 (2)	
E-selectin (ng/ml)			<0.0001
≤23	70 (7)	126 (31)	
24–37	56 (14)	47 (12)	
38–94	57 (14)	26 (6)	
≥95	13 (3)	7 (2)	
ox-LDL (mU/L)			NS
≤31.2	99 (25)	114 (29)	
31.3–41.4	46 (12)	41 (11)	
41.5–62.5	39 (9)	33 (8)	
≥ 62.6	9 (2)	8 (2)	
F2-Isoprostanes (ng/ml)			0.005
≤2.2	75 (21)	102 (29)	
2.3–4.3	5 (1)	4 (1)	
4.4–7.7	0 (0)	0 (0)	
≥ 7.8	98 (28)	65 (19)	
MDA (nmol/ml)			0.018
≤1.2	70 (18)	97 (26)	
1.3–1.5	52 (14)	48 (13)	
1.6–2.3	49 (13)	36 (9)	
≥2.4	19 (5)	9 (2)	

NS—not significant

### Independent predictor of biomarkers

To further explore the independent effect of low HDL-c on these biomarkers concentrations, multiple linear regression analyses were performed with the biomarkers as dependent variables. It was found that HDL-c is an independent predictor for IL-6 (*p* = 0.02) and sVCAM-1 (*p* < 0.03) after correcting for age, gender,ethnicity, smoking status, hypertension, diabetes, central obesity, TC, TG and LDL-c. However, using lower HDL-c concentration cutoff (≤ 0.6mmol/L for males and ≤ 0.7mmol/L for females), HDL-c was shown to been an independent predictor for MDA (p< 0.05) after correcting for age, gender, ethnicity, smoking status, hypertension, DM, central obesity, TC, TG and LDL-c (See summary in Table 5).

**Table 5:** Predictors factor for IL-6, sVCAM-I and MDA.

Variable	Predictor	Beta	S.E	Adjusted OR	95% CL		*p*-value	Constant	*R^2^*
					Lower	Upper			
IL-6 (pg/ml)	HDL-c	−0.573	0.246	−0.119	−1.056	−0.090	0.02	5.829	0.014
sVCAM-1 (ng/ml)	HDL-c	−127.922	58.431	−0.109	−242.794	−13.049	0.029	803.261	0.012
MDA (nmol/ml)	HDL-c	−0.287	0.142	−0.124	−0.568	−0.007	0.045	1.902	0.015

The model reasonably fits well. Model assumptions are met. There are no interaction and multicollinearity problem.

## Discussion

Our cross-sectional study clearly demonstrated significantly higher concentration of biomarkers of inflammation (hsCRP and IL-6), endothelial activation (sVCAM-1, sICAM-1 and E-selectin) and oxidative stress (ox-LDL and F2-isoprostane) in subjects with low HDL-c compared to NC. Similar findings were observed in a cross-sectional study by Tang et al.(2014) who reported positive association between hsCRP, sICAM-1 and E-selectin concentrations with dyslipidaemia, defined as having one or more of the following: TC ≥ 6.22 mmol/L, TG ≥ 2.26 mmol/L, LDL-c ≥ 4.14 mmol/L,HDL-c <1.04 mmol/L and/or having received treatment for dyslipidaemia in the previous 2 weeks amongst Mongolians [26]. Calabresi et al (2002) also showed that sICAM-1 and E-selectin were significantly higher in low HDL-c subjects suggesting that low HDL-c concentration promotes atherogenesis and causes acute atherothrombotic events. Furthermore, the same study illustrated a significant reduction of sICAM-1 and E-selectin concentrations in low HDL-c subjects following fenofibrate-induced increase in HDL-c[25]. Although there have been reports from various Asian countries investigating the association between HDL-c with inflammation and endothelial activation [26, 27], to the best of our knowledge, this is the first report on association of HDL-c with inflammation, endothelial activation and oxidative stress biomarkers in a Malaysian population.

It is important to note that all subjects included in this present study were not subjected to therapeutic intervention with lipid-lowering, anti-hypertensive and/or anti-diabetic medications which are potential confounding factors to inflammation, endothelial activation and oxidative stress. Lipid lowering drugs such as statins have been shown to decrease inflammatory markers such as hsCRP, sICAM-1 or sE-selectin [28] whilst anti-hypertensive and anti-diabetic medications such as glibenclamide have also been shown to reduce inflammation, endothelial activation and oxidative stress [29, 30]. HDL-c was previously shown to inhibit tumor necrosis factor-alpha (TNF-alpha) and interleukin-1 (IL-1), cytokines that induce the expression of adhesion molecules,specifically sICAM-1 and sVCAM-1 [10]. Hence, all subjects included in this present study were drug-naïve as mentioned above to avoid potential confounding factors with regard to inflammation, endothelial activation and oxidative stress, while majority of previous studies did not exclude those on these therapeutic interventions [24, 25].

With regard to biomarkers of oxidative stress, a study found significantly higher MDA concentration in patients with angiographically diagnosed coronary artery disease (CAD) when compared to healthy controls [31]. This finding of enhanced oxidative stress is in agreement with our study which showed that the concentrations of ox-LDL and F_2_-Isoprostane were higher in low HDL-c group compared to NC. However, our study showed no difference in MDA concentration between low HDL-c and NC. This may be explained by the different criteria of subject selection and the fact that MDA is not a gold standard method of assessing oxidative stress, thus giving different results [32]. These findings are also supported by previous studies which described the anti-oxidant properties of HDL-c by the inhibition of LDL oxidation through a number of apolipoproteins (apoA-1, apo-E, apo-J, apoA-II and apoA-IV) and enzymes (paraoxonase 1, platelet-activating factor acetyl hydrolase, glutathione selenoperoxidase and LCAT) [33–35]. Furthermore, it has been proposed that circulating levels of inflammation, endothelial activation and oxidative stress biomarkers may be useful in predicting the risk of developing CHD, including in those with low HDL-c concentration [9].

Our study also showed that HDL-c was inversely correlated with biomarkers of inflammation (IL-6), endothelial activation (sVCAM-I and E-selectin) and oxidative stress (F_2_-isoprostane and MDA). These results are in tandem with population studies in 2002 and 2013 which observed negative correlations between HDL-c concentration and sICAM-1, E-selectin, IL-6 and hsCRP in low-HDL-c subjects but not in those with normal or elevated HDL-c levels [25, 36]. Recent studies have revealed a strong correlation and association between ox-LDL concentration and CAD and concluded that circulating ox-LDL is a sensitive biomarker of CAD and may improve cardiovascular risk prediction [37, 38]. A study by Mascarenhas-Melo et al in 2013 showed that subjects with low HDL-c were associated with high concentrations of ox-LDL, HbA1c, TGs, non-HDL-c and hsCRP, that led to a poor cardio metabolic profile [38]. However, this present study failed to show similar correlations between HDL-c and ox-LDL. This may be attributed to the different subject selection and ELISA kits used to analyze ox-LDL.

Furthermore, our present data showed significant inverse associations between HDL-c groups (low or NC) and quartiles of each biomarker concentrations except for ox-LDL. A higher proportion of low HDL-c group was found in the highest quartile (>95^th^ percentile) of each biomarker compared to normal HDL-c group (Table 4). It has been proposed that HDL-c inhibits cytokine-induced expression of inflammatory adhesion molecules in endothelial cells [10, 25, 39]. In a study by Wadham et al (2003), it was reported that HDL-c inhibited CRP-induced expression of endothelial cell adhesion proteins [40]. A relationship between plasma concentration of HDL-c and soluble cell adhesion molecules has been reported in various studies, which revealed that HDL-c protects LDL from oxidation and decreases expression of adhesion molecules on endothelial cells including E-selectin and sICAM-1 [5, 41]. In a study of subjects with a wide range of HDL-c concentrations, it was found that plasma concentrations of soluble ICAM-1 and soluble E-selectin were significantly higher in subjects with low compared to those of average or high HDL-c concentrations [25, 26]. Therefore, these studies along with our findings further support the association between HDL-c and biomarkers of inflammation, endothelial activation and oxidative stress, reflecting the key processes in atherogenesis.

In this study, HDL-c was shown to be an independent predictor of MDA concentration at HDL-c cutoff concentrations of ≤0.6 mmol/L and ≤0.7 mmol/L for males and females, respectively, after correcting for various confounding factors such as age, gender, ethnicity, smoking status, hypertension, DM, central obesity, TC, TG and LDL-c. However, when higher HDL-c cutoff concentrations were used (<1.0 mmol/L and <1.3 mmol/L for males and females, respectively), HDL-c was also an independent predictor of IL-6 and sVCAM-I after correcting for similar confounding factors. Gomaraschi et al. (2002) have shown that HDL-c is an independent predictor for IL-6 [42] which is in agreement with the findings of this present study. In addition, we found that HDL-c was also an independent predictor for sVCAM-1 which was not seen in other studies [25, 43]. This finding suggests that in addition to anti-inflammatory and anti-oxidant properties, HDL-c also attenuates endothelial activation. Furthermore, in this present study, it has been clearly shown that HDL-c is an independent predictor of inflammation, endothelial activation and oxidative stress after correcting for confounding factors such as age, gender, ethnicity, smoking status, hypertension, DM, obesity, TC, TG and LDL-c, which were not corrected in the majority of previous studies [44, 45].

Overall, this study clearly showed that the concentrations of biomarkers of inflammation (IL-6 and hsCRP), endothelial activation (sVCAM-1, sICAM-1 and E-selectin) and oxidative stress (ox-LDL and F_2_-isoprostane) were higher in low HDL-c subjects compared to NC (Table 2). Furthermore, low HDL-c was lucidly shown in this present study to be an independent predictor for IL-6 and sVCAM-1 (*p* = 0.02 and *p* < 0.03 respectively).

It is interesting to note that in this cohort of study subjects, the LDL-c concentration was lower in low HDL-c subjects compared to NC (mean ± SD; 2.8 ± 1.1 vs. 3.4 ± 0.8 mmol/L, *p* < 0.05). LDL-c is a well-established major risk factor for atherosclerosis and CAD [46] and strongly associated with enhanced inflammation, endothelial activation and oxidative stress [47–49]. Despite lower LDL-c concentration in the low HDL-c group, the status of inflammation, endothelial activation and oxidative stress remained significantly enhanced in the low HDL-c compared to NC subjects. In addition, this present study revealed that HDL-c is a strong independent predictor of inflammation and endothelial activation, after correcting for the various confounding factors including LDL-c. Hence, these findings suggest a strong independent influence of HDL-c on inflammation and endothelial activation. According to Vergeer et al, in *in-vitro* studies indicate that HDL-c has a wide range of anti-atherogenic properties but validation of these functions in human is absent to date [50]. Although a number of animal studies and clinical trials support an athero-protective role of HDL, most of these findings were obtained in the context of marked changes in other plasma lipids [23, 50, 51]. This present study suggest a strong independent influence of HDL-c on inflammation and endothelial activation which are pivotal in the pathogenesis of atherosclerosis and CHD.

More recently, Feig et al. (2014) highlighted a controversy whether plasma HDL-c concentration reflects HDL-c function, or if HDL-c is protective as assumed. The evidence from preclinical and clinical studies have shown that HDL-c has a potential to promote regression of atherosclerosis when the levels of functional particles are increased from endogenous or exogenous sources [20, 52, 53]. These findings are in parallel with our study, implying the athero-protective role of HDL-c in independently reducing inflammation and endothelial activation. The ability of HDL-c in regressing plaque [54] and the fact that HDL-c was proven as an independent predictor of inflammation and endothelial activation in this present study suggest that the recent trial failures do not eliminate HDL-c as an atheroprotective agent but highlight the importance between HDL-c function and plasma HDL-c concentration.

## Conclusions

This present study has demonstrated that subjects with low HDL-c have higher inflammatory status, endothelial activation and oxidative stress compared to NC, which in part explains the pathogenesis of atherosclerosis associated with low HDL-c. Furthermore, we also verify that HDL-c is an independent predictor of inflammation and endothelial activation after correcting for the various confounding factors. Hence, it is important for future studies to examine the impact of newer therapies to raise HDL-c concentration on altering inflammation, endothelial activation and oxidative stress. In addition, it would also be essential to emphasize the important distinction between HDL-c function and concentration as well as to ascertain whether morbidity and mortality from atherosclerosis-related complications such as CAD associated with low HDL-c concentration are effectively reduced.
