# Correction: Low Serum High Density Lipoprotein Cholesterol Concentration is an Independent Predictor for Enhanced Inflammation and Endothelial Activation

**DOI:** 10.1371/journal.pone.0142245

**Published:** 2015-11-03

**Authors:** Wan Nor Hanis Wan Ahmad, Farah Sakri, Atiqah Mokhsin, Thuhairah Rahman, Nadzimah Mohd Nasir, Suraya Abdul-Razak, Mazapuspavina Md Yasin, Aletza Mohd Ismail, Zaliha Ismail, Hapizah Nawawi

There are errors in the author affiliations. The affiliations should appear as shown here:

Wan Nor Hanis Wan Ahmad^1^, Farah Sakri^1^, Atiqah Mokhsin^1^, Thuhairah Rahman^1,2,5^, Nadzimah Mohd Nasir^1,2^, Suraya Abdul-Razak^3^, Mazapuspavina Md Yasin^3^, Aletza Mohd Ismail^1,2^, Zaliha Ismail^4^, Hapizah Nawawi^1,2,5^

**1** Centre for Pathology Diagnostic and Research Laboratories (CPDRL), **2** Cluster for Pathology and Laboratory Medicine, **3** Primary Care Medicine Discipline, **4** Population Health and Preventive Medicine Discipline, Faculty of Medicine, Universiti Teknologi MARA (UiTM), Sungai Buloh Campus, Sungai Buloh, Selangor, Malaysia, **5** Institute for Pathology, Laboratory and Forensic Medicine (I-PPerForM), UniversitiTeknologi MARA (UiTM), Selayang Campus, Selayang, Selangor
